# Allergy as a Disease of Dysbiosis: Is It Time to Shift the Treatment Paradigm?

**DOI:** 10.3389/fcimb.2019.00050

**Published:** 2019-03-07

**Authors:** Ian A. Myles

**Affiliations:** Laboratory of Clinical Immunology and Microbiology, National Institute of Allergy and Infectious Diseases, National Institutes of Health, Bethesda, MD, United States

**Keywords:** allergy, dysbiosis, microbiome, eczema, atopic dermatitis

Attempts to strike a truce in the “nature-vs. -nurture” debate often end up framing most human disease as “a complex interaction of genes and environment.” This truce fails to distinguish the driver of disease from the risk modifier. Health care providers universally identify tobacco smoke is the root cause (i.e., “driver”) of chronic obstructive pulmonary disease (COPD) despite the known presence of associated monogenetic disorders and risk alleles (“risk modifiers”) (Hardin and Silverman, 2014). However, manuscripts reviewing ailments with less overt environmental causes, such as those covering atopic disease, default to prioritizing this dynamic in the opposite orientation. Allergic disease is frequently referred to as a “complex genetic disease” with “interacting environmental factors” (Weidinger et al., 2018), rather than a complex environmental disease with interacting genetic modifiers. To some extent, a debate about defining disease by gene-environment vs. environment-gene interactions is one of semantics. However, just as language can shape how we define a problem, so too can it influence our perception of its solution. This manuscript will center its discussion on atopic dermatitis (AD), but the commentary is applicable to allergy writ large, as well as diseases ranging from oncology to psychiatry.

Initial twin concordance studies overestimated heritability for AD to be around 75% (Weidinger et al., 2018), failing to recognize that identical twins share their environment more intensely than fraternal twins (Mayhew and Meyre, 2017). Early efforts using genome wide association studies (GWAS) to search for what these initial estimates of heritability suggested would be the causal gene were bolstered by the discovery of monogenic disorders marked by eczematous phenotypes (Lyons et al., 2015). However, many GWAS fail to find a single association; those that do, report allelic frequencies below 10% in their disease population (Bin and Leung, 2016). Of the variants implicated in AD by these individual GWAS trials, only association with *FILAGGRIN* could be confirmed by meta-analysis aggregation (Paternoster et al., 2011). While the resultant drop in sequence-specific heritability predictions to “ <20%” (Weidinger et al., 2018) does not negate the importance of identified genetic pathways [particularly interleukin (IL-) 4 and IL-13], it does suggest that the majority of patients with AD manifest disease despite genomes that are indistinguishable from healthy controls.

Often, the “missing heritability” is met with calls for better human-genomic assays, such as higher computational power combined with deeper sequencing or evaluation of epigenetic modifications. While greater technology may reveal currently undetectable interactions, it seems as if decades of advances in genomic assessment tools have only changed the working hypothesis from one stating improved genomics will uncover the causal *gene*, to one stating that improved genomics will uncover the causal *gene(s)*. This hypothesis predominates despite the burden of atopy increasing at a rate more suggestive of being driven by environmental factors (Silverberg, 2017).

In contrast with GWAS results, most microbiome publications have deployed tools of relatively low resolution to provide phyla-level differences in 16 S bacterial ribosomal RNA (Grice and Segre, 2012). For context, assays that could only provide phyla-level analysis of the animal kingdom would be incapable of distinguishing a human from a gold fish. Furthermore, additional variability in the current microbiome literature stems from differing methodical approaches such as sequencing only culturable vs. total bacteria, and/or choice of MLST (multi-locus sequence typing), ribosomal-only genomic, or full metagenomic analysis (Grice and Segre, 2012). Yet, microbiome assessments have found several disease-predictive associations between dysbiosis and atopy. Dysbiosis might be broadly defined as any composition of commensal microbes which deviates from the composition associated with health (Petersen and Round, 2014). Deviations in the microbiome of the nasal passage and lungs differentiates allergic rhinitis and asthma from healthy controls (Pascal et al., 2018). Differences in the gut microbiome associate with food allergy, asthma, and AD (Pascal et al., 2018). Differences in the skin microbiome, especially for *Staphylococcus aureus* burden, are causally associated with several features of AD pathology (Huang et al., 2017). These associations are even more pronounced if the dysbiosis is seen early in life (Byrd et al., 2017). While current associations between atopy and dysbiosis of the nasal passage, gut, and lungs are limited to bacterial signatures, additional associations between dysbiosis in commensal fungi (the mycobiome) and AD have been identified (Han et al., 2018). Contributions of commensal viruses (the virome) have yet to be elucidated for any allergic disease. However, causal links between asthma and pathologic exposure to viruses (Jartti and Gern, 2017; Kirenga et al., 2018) and parasites (Cruz et al., 2017) have been proposed.

Epidemiologic and clinical trial data further suggest that environmental and therapeutic manipulation of the microbiome can influence atopy. Northern Europeans raised on farms are relatively protected from atopy when compared to their city-dwelling cousins (Cho and Blaser, 2012; Stein et al., 2016; Pascal et al., 2018). Rural-to-urban migration increases atopy risk if the migration occurs pre-birth (maternal migration), during infancy (Rodriguez et al., 2016), or during adulthood (Tham et al., 2018). Animal models supplement epidemiologic data by directly demonstrating that parental diet can exert heritable influence over offspring immunity via the microbiome (Myles, 2014). Although the data is incomplete, there is mounting evidence that stress (either in the patients or their mother) increases the risk of allergic disease (Gilles et al., 2018). Cesarean-section delivery, absence of older siblings, early-life antibiotic use, and many other environmental factors (Kantor and Silverberg, 2017) are all strongly associated with the development of atopy and other immune-mediated diseases (Cho and Blaser, 2012; Pascal et al., 2018). Thus, while all the described environmental influencers are complex, the microbiome may partially explain the “missing heritability” through both direct passage of microbes and alteration of host epigenetics (Sandoval-Motta et al., 2017; Tost, 2018). While use of oral probiotics has shown mixed results for prevention of atopy (Cabana et al., 2017; Pascal et al., 2018), supplemental *Lactobacillus rhamnosus* enhanced the efficacy of oral immunotherapy for peanuts and milk (Pascal et al., 2018). An early-stage clinical trial using topical application of live *Roseomonas mucosa* demonstrated improvement in symptoms and associated features of AD (Myles et al., 2018), and topical coagulase negative *Staphylococci* demonstrated a reduction in the relative abundance of *S. aureus* (Nakatsuji et al., 2017).

The increased depth of microbial identification offered by metagenomics combined with metabolic pathway analysis may reveal the critical functional pathways of the microbiome that have been suggested by investigations of gnotobiotic mice (Bhattarai and Kashyap, 2016). Elucidating immunologic functions that require microbes will be just as, if not more important, as learning which specific microbes can meet those needs. Of note, improved genetic tools might uncover human variants that lack the ability to support the growth of, or properly respond to, otherwise mutualistic organisms. Therefore, paradoxically, failure to sufficiently investigate the impact of microbes on human health may be causing researchers to overlook the functional consequences of some genetic variants.

The major critique to the position of this manuscript is that current understanding of atopy deems it “obvious” that genetic contributions to allergic disease are low, while the environment is a major influencer. This sentiment is fair, and is supported by excellent recent reviews on the role of the environment in atopy (von Mutius, 2016; Gilles et al., 2018; Wikstén et al., 2018). However, the sentiment that the environment is the most important influencer of allergic disease does not appear to have been translated into the treatment guidelines. The only environmental modifications recommended by the consensus statements for AD treatment form the US (Schneider et al., 2013; Boguniewicz, 2014), Europe (Wollenberg et al., 2018), and Japan (Katayama et al., 2017) are to avoid known allergens such as animals in the home, clothing material, and foods triggers. Similarly, guidelines for rhinitis suggest avoiding those allergens known to be triggering (Dykewicz et al., 2017; Scadding et al., 2017), without any mentions of possible preventive strategies. Furthermore, a recent overview of the drugs in development for AD listed only Jak/Stat inhibitors and anti-cytokine biologics (Weidinger et al., 2018).

Under a new environment-gene paradigm, we would not only expand research into therapeutics that target dysbiosis, we would devote far more resources into examining potential nutritional interventions, stress management, and environmental modifications for the purposes of primary prevention. Current chemical safety tests limited to impacts on mammalian cells would be expanded to examine which environmental stimuli impact the microbiome and how those impacts influence human health. The original hygiene hypothesis stated that exposure to pathogens (or their molecular remnants) confers health by serving as a gauntlet for honing immunity (Strachan et al., 1996). Modern concepts around the hygiene hypothesis acknowledge that a diverse microbial exposure profile, which includes both pathogens and non-pathogens, is required for the development of a healthy immune system (Arrieta et al., 2015). Given the importance of protecting beneficial microbial communities, the potential dysbiotic side effects of pharmaceuticals would be investigated alongside current clinical adverse event assessments. This would be especially true for research into the impacts of the microbiome on early childhood development given that these formative exposures may begin *in utero* (Pascal et al., 2018).

For overall benefit to human health, the impact heralded by the dawn of the genomic era may one day only be rivaled by the onset of sanitation and vaccination. Mass scale genome searches such as the National Institutes of Health's new *All of Us* initiative will build upon *The Human Genome Project* to identify additional genetic targets and improve lives. However, while nature-vs. -nurture may be a false dichotomy, it is not akin to debating the-chicken-or-the-egg. With the mounting evidence stemming from *The Human Microbiome Project*, our paradigm must shift to incorporate an evidenced-based balance between genetic determinism, environmental influence, and microbial mutualism. Genetics will be the primary driver for some diseases while serving as modifiers for others. At least for allergy, our refusal to think outside of the helix may be limiting recognition of actionable discoveries that could provide large-scale benefit to public health.

## Author Contributions

The author confirms being the sole contributor of this work and has approved it for publication.

### Conflict of Interest Statement

The author declares that the research was conducted in the absence of any commercial or financial relationships that could be construed as a potential conflict of interest.
